# VKORC1 polymorphisms and complete resistance to vitamin K antagonists: About two cases

**DOI:** 10.37796/2211-8039.1434

**Published:** 2024-03-01

**Authors:** Ilham Benyamna, Houda El Fissi, Fadoua Bouzid, Abdelhamid El Mousadik, Najat Alif

**Affiliations:** a Department of Cardiology and Internal Medicine, Inezgane Provincial Hospital, Agadir, Morocco; b Laboratory of Biotechnologies and valorization of Natural Resources, Biology Department, Faculty of Sciences, Ibn Zohr University, Agadir, Morocco; c Department of Environment and Life Sciences, Faculty of Sciences, Ait Melloul, Ibn Zohr University, Agadir, Morocco

**Keywords:** Mutations, Resistance to vitamin K antagonists, VKORC1

## Abstract

Complete resistance to vitamin K antagonists is a rare but serious condition. It can complicate therapeutic management, especially when direct oral anticoagulants cannot be used. Some single mutations in the VKORC1 gene have been identified in patients partially or completely resistant to vitamin K antagonists. We report the cases of two women in their fifties who presented an unexplained peripheral venous thrombosis. The aetiological assessment did not show any abnormalities. Genetic testing showed that both patients had the VKORC1 5417 GG genotype. The VKORC1 3673 genotype was GG in case 1 and GA in case 2. The two patients showed complete resistance to vitamin K antagonists which required a change in treatment with favourable outcomes. Our goal is to offer optimal care guided by a literature review.

## 1. Introduction

Resistance to “vitamin K antagonists (VKA)” is uncommon, but does affect therapeutic management, especially when “direct oral anticoagulants (DOAC)” cannot be used. Resistance may be complete or partial, selective or total. Partial resistance requires the use of unusually high doses to achieve the target “International Normalized Ratio (INR)”; but complete resistance is independent of the dose. Resistance is selective when it is specific to a class of VKA, otherwise it gives way as soon as the molecule is changed [1–3]. However, total resistance occurs with all classes of VKA [4,5]. Currently, the mechanism of resistance to VKA is not fully understood. Genetic factors are a significant factor.

VKA dose variations in humans are strongly associated with single nucleotide polymorphisms in the VKORC1 gene [6,7]. We describe two cases of total and complete resistance to VKA. We have reviewed the literature to propose better management of similar cases. For information, the two cases were diagnosed among eight hundred patients on VKA followed over a period of twelve years.

## 2. Case reports

### 2.1. Case 1

A 53-year-old woman was admitted for proximal phlebitis of the left lower extremity. No contributing factor was found. She was menopausal without any hormone replacement therapy. She was hypertensive, obese, and non-smoker. The routine biological and radiological assessments did not reveal any abnormalities. Computed tomography of the brain, thorax, abdomen and pelvis was normal. Her blood group was type O+. Anti phospholipids and antinuclear antibodies were not detectable. Vitamin K dependent coagulation factors, protein S, and C were normal. Serum vitamin Ki level was high. The INR remained unchanged at 1 under a daily dose of 24 mg Acenocoumarol and 120 mg Fluindione. So she was prescribed Rivaroxaban then Dabigatran. The DOAC were quickly stopped because of the occurrence of menorrhagia and rectal bleeding without detectable lesion. The patient was prescribed Tinzaparin for 3 months, followed by acetylsalicylic acid and elastic support stockings. No event had been recorded during four years of follow-up.

### 2.2. Case 2

A fifty-year-old black woman was diagnosed with a bilateral distal pulmonary embolism. An asymptomatic distal phlebitis of the right lower limb was detected by ultrasound. She was overweight, diabetic, hypertensive and non-smoker. She had never used oral contraceptives. Her blood group was A+. There were no abnormalities found in the routine biological assessment. The radiological examination revealed a nodular goitre that was classified as grade 3 by the “**Eu**ropean **T**hyroid **R**eporting **I**maging **A**nd **D**ata **S**ystem (EU-TIRADS)”. Anti phospholipids and anti-nuclear antibodies were not detectable. Vitamin K dependent coagulation factors, protein S, and C were normal. Serum vitamin Ki level was high. The INR remained unchanged at 1 under a daily dose of 24 mg Acenocoumarol and 120 mg Fluindione. She then switched to Rivaroxaban 20 mg per day. She experienced a recurrence of venous thrombosis on the right upper limb after discontinuing Rivaroxaban for a week. Treatment was quickly resumed and the decision was made for lifelong anticoagulation. No events have been recorded during five years of follow-up since then.

Patients’ characteristics are showed in Table 1. *Genetic testing results*:

Ethical approval was obtained and both patients signed informed consent before proceeding.

DNA extraction was carried out and followed by PCR and Sanger sequencing. The VKORC1 5417G > T rs61742245 and 3673G > A rs9923231 polymorphisms were studied. The primer sequences are detailed in Table 2. We used BIOEDIT and MEGA software for the bioinformatics processing of the sequences. Genetic testing showed that both of the patients were carriers of the VKORC1 5417 GG genotype. The rs9923231 genotype was GG in case 1 and GA in case 2.

## 3. Discussion

Since 2004, a number of studies have examined the correlation between variants of the VKORC1 gene and the response to VKA [8–10]. The –1639G > A (3673G > A, rs9923231) polymorphism is the most extensively studied variant and is associated with sensitivity to VKA [11]. The AA genotype requires low doses of VKA, but the GA and GG genotypes need higher doses. The genotype of our two patients matched the last profile. The VKORC1 5417G > T (Asp36Tyr, rs61742245, D36Y) polymorphism has been associated with VKA resistance [11], but it was not found in both patients. According to research [12,13], the need for high doses of VKA has also been attributed to the presence of the CYP4F2*3, VKORC13730G > A, ABCB13435C > T, UGT1A1 (TA) n and VKORC1 369C>G (p.Ile123-Met) polymorphisms. In addition, the C20209T prothrombin gene mutation has been implicated in partial VKA resistance [14]. Furthermore, Jin et al. had recently identified ferroptosis suppressor protein 1 (FSP1), a ubiquinone oxidoreductase, as the warfarin resistant vitamin K reductase [15]. However, it should be noted that having a resistant genotype is not always associated with a resistant phenotype. Hodroge et al. demonstrated in 2012 that only six mutations out of twenty eight VKORC1 resistant genotypes correlate with the resistance phenotype [16]. Unexpectedly, we found that resistance to VKA is not mentioned in the guidelines for the proper use of anticoagulants, nor in the management of venous or arterial thrombosis [17–20]. Authors have reported cases of resistance to VKA where switching to DOAC was safe and without recurrence of thrombosis [21,22]. But there is no evidence of the effectiveness and safety of DOAC in mechanical prosthetic valves, non-valvular atrial fibrillation, and severe mitral stenosis [23]. A recent publication reported a case of resistance to VKA, which required the replacement of a mechanical valve prosthesis with a biologic one [24]. Patients should be tested for resistance before scheduled mechanical valve replacements to avoid redoing surgery. One may wonder whether genetic tests should be conducted prior to any valve surgery. The identification of a genotype related to the resistance phenotype may be of interest if these tests are available. A recent Korean study did not show any clear advantages of performing these tests in patients with aortic valve prosthesis in terms of the “time in the therapeutic range (TTR)” [25]. So, it seems that there are multiple factors influencing the response to VKA. Other studies should be conducted. In the short term, we may need to try new drugs that are less influenced by genetic and environmental factors. Ticarfarin looks promising [26]; FXI/FXa inhibitors are currently under investigation [27]. We can expect to see drugs that target FSP1 in the future. In conclusion, managing patients with total and complete resistance to VKA is a real challenge, especially in situations where there are no other therapeutic options. It is necessary to develop new drugs and promote research to achieve a better understanding of the mechanisms of resistance. Patients should be tested for VKA resistance before scheduled mechanical valve replacement surgeries.

## Figures and Tables

**Table 1:** Patients’ characteristics

Parameter	Case 1	Case 2
ABO group	O+	A+
BMI	32	29
High BP	Yes	Yes
Diabetes	Yes	No
APLA	ND	ND
Hb g/dl	13	12
WBC/mm3	5430	4689
RBC/mm3	4 × 106	5,2 × 106
Platelets	155000	261000
ANA	ND	ND
Creatinin	9 mg/l	11
Protein	67 g/l	73
Calcemia	93 mg/l	88
Uric acid	46 mg/l	57
CRP	7 mg/l	10
LDH	170U/l	190
LDL C	1 g/l	1.7
TG	0,8 g/l	2.3
GOT	17U/l	20
GPT	18U/l	23
Na	135mEq/l	142
K	3,9 mEq/l	4.3

(BMI: body mass index, BP: blood pressure, APLA: anti phospholipid antibodies, ND: not detectable, Hb: haemoglobin, WBC: white blood cells, RBC: red blood cells, ANA: antinuclear antibodies, CRP: c reactive protein, LDH: lactc dehydrogenase, LDL C: LDL cholesterol, TG:triglycerides, GOT and GPT: transaminases, Na: sodium, K: potassium).

**Table 2:** Primer sequence.

VKORC1	F: 5′- AACCTGGAGATAATGGGCAGCA- 3′
5417G > T	R: 5′-ACACCGATCCCAGACTCCAGAATA-3′
VKORC1	F: 5′ ATCCCTCTGGGAAGTCAAGC 3′
3673G > A	R: 5′ CACCTTCAACCTCTCCATCC 3′
